# Should Sabbath Prohibitions Be Overridden to Provide Emotional Support to a Sick Relative?

**DOI:** 10.5041/RMMJ.10250

**Published:** 2016-07-28

**Authors:** Chaya Greenberger, Pnina Mor

**Affiliations:** 1 Dean, Faculty of Life and Health Sciences, Jerusalem College of Technology, Jerusalem, Israel; 2 Chair, Department of Nursing, Jerusalem College of Technology, Jerusalem, Israel; 3 Department of Nursing, Jerusalem College of Technology, Jerusalem, Israel; 4 Shaare Zedek Medical Center, Jerusalem, Israel

**Keywords:** Emotional support, halacha, life-saving, overriding Sabbath laws

## Abstract

**Background:**

There is a consensus among the halachic authorities that life-saving actions override Sabbath prohibitions. They are painstaking in securing that the sanctity of the Sabbath is maintained but that not a single life be lost.

**Objective:**

This manuscript examines if and when a relative’s presence at the bedside of a seriously ill individual is potentially life-saving against the backdrop of the scientific literature. It specifically addresses the permissibility of traveling in a motorized vehicle, generally prohibited on the Sabbath, to be with one’s relative in hospital for the provision of emotional support.

**Methods:**

Discourse of the halachic issues in the context of the scientific literature.

**Results:**

Stress, mental or physical, has been determined as a potentially life-threatening condition in many disease entities. The literature attests to both the patient’s and the professionals’ perception of the curative potential of the presence of loved ones by advocating for the patient and relieving stress in the hospital experience. Emotional support from a loved one is perceived by some patients as vital to survival. There is halachic consensus that a patient’s perception of the emotional need for a relative’s presence is sufficient to permit overriding rabbinic prohibitions. Torah prohibitions, which may be overridden for medical needs, may be overridden for emotional support, providing a health professional or family member attests to the fulfilment of this specific need as diminishing the danger to the patient’s life. In certain cases, the latter contingency is unnecessary.

**Conclusions:**

Emotional support has an impact on the patient’s health status; the degree to which its impact is strong enough to save life is still being studied. As more data from scientific studies emerge, they may be relevant to sharpening the halachic rulings with respect to the issue at hand.

## INTRODUCTION

There is consensus among halachic authorities that life-saving actions override Sabbath prohibitions. This manuscript examines if and when a relative’s presence at the bedside of a person in a life-threatening condition (*choleh shyesh bo sakana*) is considered potentially life-saving. Specifically, it addresses the permissibility of traveling in a motorized vehicle, generally prohibited on the Sabbath, to be with one’s seriously ill relative in hospital for the provision of emotional support. The relevant scientific literature prefaces the halachic discourse in order to illustrate its reflection of the halachic rulings.

It is remarkable that, traditionally, hospitals restricted or even barred visiting severely ill patients. Reasons for these prohibitions included the fear that visitors presented both a threat to patients (via the risk of infection and the increased stress of “hosting”) and that medical staff considered them a hindrance to patient care.^1^–^3^ It is now known that the presence of relatives by the bedside of a patient contributes to maintaining and even improving the physical and mental health of the patient due to the support, particularly the emotional support, that they provide.^4^–^12^ In this regard, clinical practice guidelines of the American Association of Critical Care recommend open visitation for family members,^13^ and the American Association of Critical Care Nurses delineates around-the-clock support by kin as expected practice in intensive care units^13^,^14^ as it diminishes anxiety, enhances safety and security, and minimizes complications.

## THE ILLNESS EXPERIENCE AND THE DETRIMENTAL EFFECTS OF PSYCHOLOGICAL STRESS

Acute illness is an assault on physiological homeostasis, but also an existential threat, as an individual finds himself/herself in strange surroundings, often helpless, in pain, and tense with respect to the unknown future. Anxiety and depression are manifestations of this experience.^15^ The combination of these factors creates stress, a condition in which strain can exceed the ability of the individual to adapt, causing distress.^16^,^17^ Hans Selye identified a three-stage common physiological response to stress—physical or psychological—coining the term “general adaptation syndrome” (GAS): alarm, resistance, and exhaustion.^18^ Lipp (in Lucinda et al.) identified an additional stage of semi-exhaustion.^19^ In the alarm stage, stress launches the secretion of adrenaline, accompanied by psychological arousal. This typically triggers a therapeutic increase in blood pressure and pulse rate and stimulates immune activity. However, prolonged stress or short-lived stress of a large magnitude is detrimental. As the individual enters the stage of resistance, cortisol is secreted in an attempt to re-establish homeostasis, and anxiety is pervasive. This may increase vulnerability to infection, prevent or delay surgical healing, and continue to tax the heart. The quasi-exhaustive stage is characterized by the beginnings of general organ deterioration, with the exhaustive stage resulting in depression and organ failure. Research conducted by Lucinda et al. (*n*=42) found that 72% of patients were experiencing stress four days post-acute myocardial infarction, 71% in the resistance stage.^19^

A large body of research has confirmed the potentially negative impact of stress. As it is beyond the scope of this article to address all the research linking psychological stress to negative outcomes in illness, studies most relevant to the subject at hand have been chosen. Moser et al.,^20^ for example, found the degree of anxiety in the first few hours following an acute myocardial infarction to be a significant predictor of complications such as fatal arrhythmia and excessive life-threatening clotting, after controlling for other variables (e.g. the size of the infarction, side effects, and previous infarctions). This is remarkable as it lends evidence to the detrimental effects of stress on the cardiac muscle in the initial “constructive” stage of “alarm reaction.” The heart has unique vulnerability as it labors hard from the start to nourish the stress response. In this regard, Krantz et al.^21^ reported extreme anger as an immediate trigger of myocardial infarction and found general psychological stress a trigger for acute cardiac events in illness situations, including arrhythmias and sudden death. Similarly, Huffman et al.^22^ reported a correlation between severe anxiety and depression and sudden onset of excessive clotting in cardiac patients.

An acute life-threatening stress response prevalent in hospitalized patients is delirium. The condition develops through the combined presence of physiological factors such as infection and fluid/electrolyte imbalance, coupled with environmental ones such as stimulation overload and isolation from loved ones.^23^ Patients display delusionary confusion and disorientation, becoming either hyper- or hypo-actively unco-operative, dysfunctional, and potentially harmful to themselves and their surroundings. Ryan et al.^24^ reported the point prevalence of delirium in hospitalized patients at 20%, with some studies reporting percentages of delirium in intensive care units to be as high as 70%. It is the leading complication of hospitalization for older adults.^25^–^27^ Delirium is associated with serious negative consequences, including increased morbidity and mortality, with an 11% increase in mortality for every additional 48 delirious hours.^24^ Stressful psychological concomitants of acute illness can remain a threat to life beyond the acute phase of illness, resulting in varied morbidities generally and post-traumatic stress specifically.^28^ The Diagnostic and Statistical Manual of Mental Disorders (DSM IV) now categorizes acute illness as a potential antecedent of post-traumatic stress disorder (PTSD)^29^,^30^ in light of the accumulated evidence regarding the possible delayed life-threatening sequels of illness on both physical and psychological health. In a prospective study, 6 (14%) out of 43 patients mechanically ventilated in intensive care units developed severe PTSD at 6 months’ follow-up.^31^ In a meta-analysis conducted by Edmonson et al.,^32^ PTSD was found to be both prevalent after acute cardiac illness and associated with future cardiac events and higher subsequent mortality, with a 55% increase in risk, after controlling for other risk factors. Spindler and Pedersen^30^ reported perceived severity of the illness, rather than the objective illness severity, to be a better predictor of PTSD; Guler et al. found feelings of helplessness to be especially predictive. 33

Symptoms of post-traumatic stress (PTS) can also manifest themselves while an individual is still in the early recuperative stages of illness. Talisayon et al.^34^ reported that PTS symptoms were present in more than one-third of the critically ill within 1 week post-hospitalization. In intensive care unit (ICU)-ventilated patients, rates of PTS symptoms were reported at 27%, 24%, and 12% in different reports,^34^ whereas for post-operative cardiac surgery patients the percentage was reported to be 14.7%.^35^

## SOCIAL SUPPORT MODERATES STRESS AND ITS OUTCOMES

Lazarus and Folkman^36^ identified social support as one of the critical resources available for enduring stress, including that of illness and post-trauma. Research over the last few decades points to social support as a significant factor in decreasing morbidity and mortality. The mechanisms include a direct decrease in physiological reactivity of the cardiovascular and neuroendocrine systems^37^,^38^ and an indirect positive impact on coping.^39^

Social support is often divided into emotional, informational, and practical support, the latter two facilitating decision-making and effective health behaviors.^17^ Evidence, however, points to perceived emotional support—best provided by loved ones—as most influential in stress reduction by reassuring a person that he or she is a valuable individual about whom people care. Meaning, purpose, and a sense of worth and belonging which goes to the core of human existence are nourished by emotional support.^17^,^40^ In this vein, Herlitz et al.^41^ reported that, among 1,290 patients who underwent coronary artery bypass surgery, ratings of the statement “I feel lonely” predicted survival at 30 days, even after controlling for preoperative factors known to increase mortality.

## FAMILY PRESENCE AT THE BEDSIDE

The critical care environment, intensively technological, diminishes personhood and arouses feelings of alienation.^42^,^43^ Families provide identity, security, and comfort, while significantly reducing anxiety in this intimidating environment.^44^–^52^ Patients reported that physical and verbal contact with family members during invasive and resuscitative procedures was a “healing force” that enabled them to cope more effectively with stressful experiences.^47^,^53^,^54^ In a study of patients undergoing liver transplants, family presence was cited as the most important source of support,^55^ with 35% requesting their presence during the actual transplant procedure. Fredriksen and Ringsberg’s review,^56^ moreover, points to separation from loved ones as itself a cause of stress.

With regard to specific stress responses to illness, family integration into the continuous care of patients has been found to be protective against the development and exacerbation of delirium.^57^,^58^ Martinez et al.^59^ found that family intervention reduced incidence of delirium by 58%, and it is now part and parcel of the National Institute for Health and Care Excellence (NICE) treatment guidelines for delirium. Similarly, a significant body of research has pointed to the presence of significant others as a possible buffer against the development of PTSD subsequent to traumatic stress.^61^–^63^

Many qualitative studies reflect the critical importance of family presence. In research by Mylen et al.^64^ of former neurosurgical intensive care unit patients, respondents reported how family members infused them with a feeling of security, sense of person, purpose, and motivation. “It was a lot of energy like … you know healing, in the words of one of the patients … to have family around” (p. 45). It was a “reminder” of belonging that gave patients motivation to recover.

In a similar vein, Alpers et al.^65^ found the family to have great impact on bolstering the patient’s inner strength “to go on living” (p. 155), an expression also used by patients in Nygren’s study.^66^ Another patient put it this way: “Just lying there … not moving … I wouldn’t know how I would have been today, if she [her mother] hadn’t been there.”^67^ In the study undertaken by Bergbom and Askwall,^44^ although acknowledging the importance of instrumental assistance given by relatives, patients singled out the moral support they received as “restoring to life.” Similarly, Wang et al.^68^ interviewed patients shortly before they were released from the ICU; one of the most momentous statements was: “My family gave me courage to persist; I might have given up without their backup” (p. 187). The latter two statements clearly indicate that in the patient’s mind a relative’s emotional support is life-sustaining.

It is remarkable that mere presence has been singled out in various studies as the most critical component in family support.^45^ One patient in the study by Twibell et al.^69^ said the following: “It’s important for my family to just be there: They don’t have to do anything. We can just look over at each other … I knew when I saw them that I mattered to them. They hadn’t forgotten or given up on me. I want them to be here with me” (p. 111). A patient interviewed by Wahlin et al.^70^ similarly commented: “It feels safe when you’re lying there, to have someone from the family with you … I don’t have the energy to talk, but he understands that. He just sat there and held my hand” (p. 374). In terms of quantitative research, Rotondi et al.^71^ interviewed 150 patients following their hospitalization in an intensive care unit, where they were connected to a respirator for more than 24 hours. Of the 41 patients who recalled missing their relatives, 31 reported that this affected them significantly. Out of 38 patients who remembered a feeling of isolation, 28 reported that this noticeably distressed them. Novaes et al.^72^ interviewed 50 intensive care unit patients in order to determine their sources of stress. The data were collected via the Intensive Care Unit Environment Stressor Scale, a 40-item Likert scale evaluating physical and mental stress. The patients recorded severance from the family as a source of stress.

Cornock^73^ interviewed 71 intensive care unit patients who had been connected to a respirator, as well as 71 nurses from the unit. The two groups were asked to report on three characteristics of the 50 characteristics in the Environment Stressor Scale that constituted the most significant sources of patients’ stress. Eight of the patients included missing their spouse among one of the first three choices, and seven of the patients included time limitation on visits as one of the top three. Nurses similarly graded these two characteristics at the same level as patients, lending professional validity to patients’ perceptions.

In Williams’s study,^48^ a total of 67 nurses reported their observations of patients and their respective families. One nurse related her attempt to wean a patient off a respirator in the presence of a relative. She noted that the patient relaxed more quickly in his presence. Another described her success in weaning down pressure delivered by CPAP (continuous positive airway pressure to support breathing) in the presence of a relative as the patient’s breathing became more effective.

In a randomized trial conducted regarding restricted visitation policy (RVP), Fumagalli et al.^74^ studied the influence of relatives’ visits on the medical state of patients. The researchers compared two similar socio-demographic groups of patients (*n*=111 and 115) with comparable clinical characteristics, who were hospitalized in the intensive care unit for an extended period of time. The number and length of visits was restricted for one group, while the second group benefited from an unrestricted visitation policy (UVP). Compared with the unrestricted group, the patients with restricted visitations had a 2-fold greater risk of major cardiovascular complications, particularly of pulmonary edema or shock, but also, although not significantly, of arrhythmias and cardiac rupture. The unrestricted group was associated with a greater reduction in anxiety score and a significantly lower increase in thyroid-stimulating hormone from admission to discharge. Furthermore, the mortality rate among those whose visits were not restricted was 1.8% compared to 5.2% in the groups whose visits were restricted. In another analysis of 156 patients (RVP, *n*=80; UVP, *n*=76) being treated for myocardial infarction these researchers compared the Killip class distribution (a stratifying of risk criteria) between admission to and discharge from the ICU. The Killip level improved by 58.8% among those whose visits were unrestricted, while only 3.4% of them deteriorated. In those patients whose visits were restricted, only 26.7% improved and 6.7% deteriorated. The clinical differences between the groups were attributed to the reduction in stress and anxiety arising from the unrestricted visits. Researchers using intracranial pressure to measure changes in stress reported a decrease in pressure following relatives’ visits.^74^,^75^ Others, using pulse rate and blood pressure, similarly observed a decrease in both indicators following visitation.^10^,^48^

## RESEARCH SUMMARY

Ample evidence points to the importance of family support and presence in alleviating stress in illness and preventing or diminishing its negative sequels. Relationships between emotional support and stress/illness factors have been assessed both qualitatively, by interviewing patients and professional caregivers, and quantitatively, via objective measures. In the former, interviewees in different studies have repeatedly used words with roots “life” and “heal” in describing what family presence means. In the latter, many objective measures—vital signs, cardiac and neurological indicators, psychological and physiological morbidity, as well as mortality—vary positively to different degrees with emotional support.

## THE HALACHIC DISCOURSE: TENDING TO THE MEDICAL AND EMOTIONAL NEEDS OF THE SERIOUSLY ILL ON THE SABBATH

Against the backdrop of this research, we address the following case: Rabbi Zalman Nehemiah Goldberg^76^ was asked (in 1986) to render a halachic decision with respect to an individual hospitalized after open-heart surgery who suddenly felt unwell and requested that his son travel on the Sabbath in order to be at his bedside. As this involved overriding a Torah prohibition, Rabbi Goldberg permitted fulfilling the request only if the son were certain that his visit would have life-saving implications. It is implicit in this ruling that the presence of a loved one for emotional support may, in certain cases, be potentially life-saving; however, the patient himself is not relied upon to be the judge.

Rabbi Goldberg’s stipulation is surprising in the light of other cases in which the patient’s subjective appraisal of his condition as being potentially life-saving suffices to override Torah prohibitions, since “the mind knows the suffering of the soul” (Proverbs 14:1). Accordingly, Rabbi David ben Solomon ibn Zimra (1479–1573), also called Radbaz, ruled that we comply with a patient who claims that he or she needs certain medications on the Sabbath even if the doctor considers that there is no need, as long as the doctor confirms the medication will do no harm.

This responsum of Radbaz is cited by the Tzitz Eliezer^77^ in connection with the halachic question addressed in this manuscript. The latter distinguishes between a patient’s request for medical treatment on the Sabbath, in which case one may override Torah prohibitions even without the doctor’s consent, and the patient’s request for a relative to come to stay by his or her beside, for which purpose these prohibitions may not be overridden. The first impacts directly on the healing process, whereas the second only improves the patient’s emotional state. However, continues the Tzitz Eliezer, if a doctor, an authoritative professional, were to stipulate that the absence of the relative could potentially endanger the patient (as Rabbi Goldberg ruled with respect to the relative), the prohibitions need be overridden as with regard to any action related to healing. Rabbi Epstein, the author of Aruch Hashulchan,^78^ and Rabbi Hadaya, author of Yaskil Avdi,^79^ likewise rule that if a doctor affirms that not granting a sick individual’s request to send for his or her relative would put his or her life in danger, this must be regarded as equivalent to medical treatment, and the Torah prohibitions of the Sabbath must similarly be overridden.

With respect to the impact of perceptions on healing, we turn to Maimonides’ *Hilchot Avodah Zarah*^80^ in which he permits so-called “whispering” (a technical term for a type of sorcery alleged to cure). While this is, in his opinion, Torah-forbidden as a superstitious practice (and hence akin to idolatry), at the insistent request of a dangerously ill individual, it is permitted even on the Sabbath, in order to prevent extreme mental anguish. This ruling is made despite the fact that Maimonides himself is convinced there is no cure in this. Halachic standing is given to the patient’s belief in the healing power of certain actions, even when the belief is mistaken.

The author of *Nefesh Hayyah*^81^ cites Maimonides’ attribution of halachic status to subjective perceptions as support for his position regarding a case similar to ours. In the case of a dangerously ill individual who expresses a longing to see his relative, *Nefesh Hayyah* was asked whether a relative may override Torah prohibitions in order to be at the patient’s bedside. The author gives standing to the request but iterates that overriding these prohibitions would, in addition, necessitate some objective evidence regarding the curative potential of the relative’s presence. The assumption may be that the heightened emotional state of the sick individual might bring him to request his relative’s presence, even if he himself does not truly perceive the latter’s absence to be life-threatening. Regarding treatment, however, his perceptions, as we have seen, are assumed to be genuine. A more straightforward possibility is that not fulfilling the individual’s request regarding treatment is deemed by *Nefesh Hayyah* to be more detrimental than a respective decision regarding the request for a relative’s presence.

Rabbi Shmuel Wosner^82^^(Part 8:65)^ concurs with this ruling, differentiating between a seriously ill individual and a woman in the post-partum state. With respect to the latter, because the birth experience is uniquely laden with emotion, no additional attestation is needed to confirm that emotional well-being has life-saving implications. Therefore, Rabbi Jacob ben Asher, the author of *Orach* Chayim,^83^ stipulates that a light may be lit for her even if she is blind, so that she should not be afraid. With regard to the sick individual, the rabbi ruled that a doctor must attest to the life-threatening potential of the emotional stress (and thus the vital need for emotional support).

### Torah Versus Rabbinical Prohibitions on the Sabbath

According to Rabbi Elijah ben Shlomo Zalman, known as the Vilna Gaon (Genius of *Vilnius*),^84^ one may send a non-Jew on the Sabbath to arrange for the relative of a seriously ill individual to travel immediately after the Sabbath, to be at his bedside. Here again, only a minor rabbinic prohibition is being overridden, making it permissible in order to relieve mental anguish. *Mishna Berurah*^85^ extends the removal of rabbinic prohibitions beyond that of just asking a non-Jew to be an informant to hiring a non-Jewish runner. *Shulchan Aruch Shel HaRav*,^86^ however, rules that as the presence of a relative does not affect any real medical recovery, but merely eases emotional suffering, rabbinically forbidden actions may only be undertaken by a non-Jew.

A somewhat different case is presented in the responsa of the *Shoel U’Meshiv*,^87^ cited also by Rabbi *Yisrael* Matisyahu Auerbach,^88^ in which a man hears that his sick wife has become stricken with acute anxiety and is in a village where nobody knows her. The *Shoel U’Meshiv* rules that the husband may ride there by horse on the Sabbath (a major rabbinic prohibition, as it is being performed by a Jew). He reasons that the wife will certainly benefit from her husband’s arrival, and this is a case of “possible life-saving” (*safek pikuach nefesh*) which overrides the laws of the Sabbath. The *Shoel U’Meshiv* does not explain his position.

The author of *Helkat Yaakov*^89^ perceives the life-saving elements of the husband’s presence as nested in the overall benefit that the woman receives; apart from easing her mind he will provide practical assistance (i.e. safety measures, hygiene) which justifies overriding major rabbinic prohibitions (and, as we have seen earlier, Torah prohibitions). If this is an accurate interpretation of the *Shoel U’Meshiv*, it cannot be deduced from this that mere emotional support alone would be sufficient to permit a Jew to ride a horse on the Sabbath. Furthermore, a patient is not alone in a hospital as staff tends to both practical and emotional needs.

The responsum of *Migdal Hashen*,^90^ cited by Rabbi Waldenberg,^77^ relates specifically to the distinction between emotional support and attendance to the patient’s practical needs. He discusses the case of a sick individual who sends a letter to another town urgently requesting a doctor as he is in danger. He rules that a Jew may travel (on a wagon, a minor rabbinic prohibition) on the Sabbath with the doctor in order to ensure that the doctor arrives as soon as possible. He raises the possibility that the Jew may even be permitted to travel alone (a major rabbinic prohibition) as it is permissible to light a lamp for a woman after childbirth, even if she is blind, to settle her mind in case she is afraid, and that her fear may endanger life.

Unlike the Shevet Halevi,^82^^(Part 8:65)^ who attributes permission to light a lamp for a blind woman post-partum to ease her mind overall, *Migdal Hashen* attributes it specifically to allaying her fear regarding the impact of the darkness on the quality of the treatment and therefore equates her with the seriously ill individual. For concerns of treatment, even a Torah prohibition is overridden for both these cases. *Migdal Hashen* equates this case to permitting a relative to travel with the doctor to ease the patient’s fear that the doctor may not look after him properly. It must be stressed that the action required is *directly* connected with medical needs. On the other hand, the presence of a relative to ease the emotional distress of being alone is not designated a priori by the author of *Migdal Hashen* as potentially life-saving. It is remarkable that there is a consensus among halachic authorities regarding the obligation to override Torah prohibitions in order to provide information to the health care provider and advocate for the patient. *Shevet Halevi* (8:68) stipulates that it is always mandatory for a family member to accompany an unconscious patient, as he is certainly incapable of human interaction.

### Mental Anguish May Be Life-threatening

Rabbi Wosner^82^^(Part 50:71)^ cites examples of situations that are life-threatening in and of themselves, specifically because they cause extreme mental anguish. The Babylonian Talmud states: “If a child is locked behind a door on the Sabbath the door may be broken to bring him out.”^91^ Rabbi Wosner similarly rules that Torah prohibitions may be overridden to free a trapped, panic-stricken adult. Rabbi Neuwirth^92^^(Part 32:15;Part 41:27)^ likewise perceives relieving intense fear as a sufficient reason for overriding Torah prohibitions for a seriously ill individual who is afraid of the dark.^92^^(Part 32:63)^ In contradistinction to these cases, halacha could argue that a distraught patient who calls for his relative is not always in an acute state of panic and, what is more, the relative’s presence does not neutralize the fear as the illness is ever-present.

A number of additional halachic authorities, however, consider mental anguish as potentially life-threatening with respect to the Torah prohibitions. The author of *Pri Megadim*^93^ categorizes extreme mental anguish as life-threatening, for which even Torah prohibitions may be overridden. *Minchat Yitzhak*^94^ cites further authorities, namely the *Levush*,^95^ *Tosefot Shabbat*,^96^ and *Levushi Srad*,^97^ who regard mental anguish as a potentially life-threatening situation and also contend that it may be eased by the presence of a significant other.

Rabbi Ovadia Yosef^98^ ruled that a seriously wounded soldier who requests a relative’s presence for the sole purpose of easing his mind is similar to a woman after childbirth; Torah prohibitions may be overridden in order to fulfill his request, and there is no need to obtain anyone else’s opinion regarding the matter’s urgency. In addition to establishing mental anguish as life-threatening in certain cases, Rabbi Yosef also establishes that the presence of a loved one is potentially life-saving through easing the anguish. Perhaps a victim of terror would also fall into this category.

It is possible that Rabbi Neuwirth may have modified his stand regarding the permissibility of a relative accompanying an individual to hospital on the Sabbath. In the second edition of *Shemirath Shabbath*,^99^ he permitted riding in the vehicle which is transporting a loved one to hospital, a minor rabbinic violation (if at all). In the second edition of *Nishmat Avraham*,^100^ Rabbi Neuwirth is quoted as permitting driving even in a separate vehicle, but only for the purpose of providing practical assistance in hospital, such as giving a medical history. In the third edition of *Shemirath Shabbath*,^92^^(Part 40:72)^ Rabbi Neuwirth clearly states that riding in a separate vehicle is permissible, both in order to be present for emotional support and to provide information for his/her relative upon arrival at the hospital. This ruling implies equal life-saving potential in both roles—practical and emotional.

### Additional Special Cases

*Shemirath Shabbath*^92^^(Part 32:26)^ rules that one may be lenient even regarding Torah prohibitions with respect to a patient whose chance of recovery depends on his or her emotional state. The example offered is of an individual predisposed to depression who might behave dangerously with regard to himself or others if he perceived that he was not properly being cared for.

No specific halachic ruling has been found by these authors regarding relieving emotional stress levels by traveling to the patient’s bedside for individuals who are suffering from acute medical conditions especially sensitive to emotional status, such as a myocardial infarction. Such stress can, as the literature points out, be immediately life-threatening, finding expression in potentially fatal arrhythmias, excessive blood clotting, spiking high blood pressure, and respiratory distress. They also harbor the seeds of potential threat to life at a future time, which, according to some halachic authorities, may warrant overriding even Torah prohibitions.^101^

Barring fatality, there is also the real possibility of permanent mental deterioration, seriously impinging upon the ability to live a Torah-observant life. In this case, the principle of overriding one Sabbath in order to enable the observance of many more in the future might become applicable. Although emotional support of significant others is part of the preventative protocol for potentially fatal conditions of delirium and post-traumatic stress, it must be kept in mind, however, that social support is only one interventionary measure within a complex treatment protocol, and its therapeutic weight is not readily assessable.

Beit Yehudah^102^ cites a case of a dying individual who lay in a dark house and ruled it permissible to light a lamp on the Sabbath (a Torah prohibition) so that he might see his relatives, thereby soothing his mental anguish. Rabbi Mordecai Gutman^103^ perceives this ruling as being based on respect for a human being who is made in the Divine image, a supreme need for which Sabbath prohibitions may be overridden. One might argue, more simply, that in this case seeing his relatives might allay his anxiety and thus lengthen his life even if only for a short period. The professional literature points out that fear of dying “alone” can cause worse distress than the fear of death itself. The question of traveling to be beside an individual on his deathbed on the Sabbath was not herein specifically addressed. Nevertheless, it would seem to be no less important than lighting a lamp to enable the individual to see relatives known to be present.

### What Are Considered “Needs” of a Seriously Ill Individual?

*Shulchan Aruch*^104^ and Maggid Mishneh^105^ are of the opinion that all the needs of a seriously ill individual may be met on the Sabbath in a fashion similar to a weekday (e.g. overriding Sabbath prohibitions) even if they are not essentially life-saving. This has relevance to our discourse since if all needs may be met, this would also include the presence of a relative at the bedside, even if it were not, per se, a life-saving action. Other authorities such as Rashi and the Geonim permit only those actions that actually mitigate danger.

Perhaps there is no real disagreement. Radbaz (cited by Rabbi Waldenberg^77^) delineates that the type of need of a sick individual which is permitted is any need that has a life-saving aspect to it, even if only indirectly. Since the patient is already in danger, the range of needs should be expanded to include those with even a remote possibility of impacting on life-saving. Clearly, however, as Rabbi Shlomo Zalman Auerbach remarks (as cited by Rabbi Avraham S. Abraham^106^), the line of demarcation would not include delivering a newspaper or turning on a radio, which would certainly not be permissible.

Rabbi Wosner^82^^(Part 8:71)^ holds a similar position: regarding a *choleh shyesh bo sakana*, it can never be fully known what can have a detrimental impact on his condition. Even if refraining from the fulfillment of a need does not immediately increase danger, it might possibly weaken the individual over time and decrease his ability to overcome his illness. This seems to expand the time frame of *pikuach nefesh*; even future danger warrants overriding Torah prohibitions. Despite this categorization, it will be recalled that Rabbi Wosner forbids relatives from breaching Torah laws in order to be at the side of *choleh shyesh bo sakana*, the initial assumption still being the lack of correlation between a relative’s presence and *pikuach nefesh*, unless proven otherwise.

Rabbi Asher Weiss^107^ goes a step further. While it is not possible to assess what will cause a seriously ill individual to succumb to death, anything that is related to a cure, affects healing, or provides an improved feeling of well-being is to be considered a life-saving act, similar to easing the mind of the woman in confinement. Although no specific ruling has been given regarding our case, it is possible that Rabbi Weiss would permit it.

Rabbi Moshe Farbstein’s approach is similar.^108^ With respect to the seriously ill individual, the assessment of what is considered to be life-saving is made at a different level. It is clear from the medical literature that a patient who has a life-threatening condition does not have the mental and physical reserves that non-threatened patients have. Therefore, when considering his or her needs, even those remotely related to healing must be met.

Rabbi Farbstein relates to another element that affects the definition of life-saving, namely public opinion. That which, in the opinion of the public, is considered necessary for life-saving, even if in fact the connection is far-fetched, must be considered as life-saving for halachic purposes, and Rabbi Auerbach^109^ comments likewise. Rabbenu Tam^110^ considers that a dog bite is, objectively, very far from dangerous to life, but since public opinion considers it dangerous, it must be considered as such, and the Sabbath laws may be overridden in such a case.

## CONCLUSION

There is clear evidence in the literature regarding the detrimental effects of stress and the positive impact of a relative’s presence on the process of recovery through alleviating stress. This has spurred widespread policy changes regarding visitation. Although there is a dearth of randomized controlled trials, there are empirical studies that lend substantial evidence to stress reduction in the presence of relatives, with subsequent decreases in potentially fatal complications in unstable patients.

From a subjective perspective, patients report the importance of a relative’s presence using terms relating to life-saving and survival. According to some halachic authorities, patients are not solely reliable reporters when it comes to their emotional needs. However, relatives and nurses have also attested to the importance of the relative’s presence for such instances as being weaned off ventilating devices and reducing anxiety. Halachic authorities refer to physicians as the authoritative health professional; perhaps as nurses continue to become more autonomous they will also be considered authoritative in this regard, especially as they are often the health care providers most attuned of all to the patient’s emotional state and needs. The public’s perception regarding what constitutes danger also has halachic validity, as Rabbi Farbstein has pointed out.^104^ It is therefore important to continue to follow the professional literature and public opinion regarding the impact of stress, the impact of family presence, and the connection between the two. Further studies regarding these phenomena may affect future halachic rulings.

Halachic authorities are painstaking in their rulings in order that the sanctity of the Sabbath may be maintained, but that not a single life should be lost as a result. There is a delicate balance to maintain, and we have seen shades of opinions. With respect to traveling on the Sabbath in order to be with a hospitalized loved one for the sole purpose of giving emotional support, most authorities only permit overriding rabbinic prohibitions if a doctor attests to it being a matter of *pikuach nefesh*, although as we have seen, there are some important exceptions regarding the place of family support in illness as reflected in the literature. These are special cases in which emotions categorically play a dominant role in life-saving.

In reality, however, when a relative is summoned to a patient’s bedside on the Sabbath, his/her arrival may be vital for both medical and emotional needs. In this regard, Rabbi Mordechai Halpern,^76^ after surveying a broad range of relevant halachic opinions, concludes that, when actually confronted with the situation, a loved one must travel to the scene without hesitation and without speculating which of the two needs the presence is apt to meet and to what degree. The overall situation, he iterates, is clearly one of *safek pikuach nefesh* for which “one who responds speedily is to be praised and one who hesitates should be rebuked.”^111^

## GLOSSARY

Halacha: The corpus of Jewish religious law rooted in the Bible and continually being expanded by its designated authorities
*Choleh shyesh bo sakana*: An individual whose state of health endangers his life
*Safek pikuach nefesh*: A situation in which there is a potential danger to human life, which necessitates taking immediate action

## Abbreviations

ICU: intensive care unit
PTS: post-traumatic stress
PTSD: post-traumatic stress disorder
RVP: restricted visitation policy
UVP: unrestricted visitation policy
